# Expression of Lewis y antigen and integrin αv, β3 in ovarian cancer and their relationship with chemotherapeutic drug resistance

**DOI:** 10.1186/1756-9966-32-36

**Published:** 2013-06-01

**Authors:** Jian Gao, Zhenhua Hu, Dawo Liu, Juanjuan Liu, Chuan Liu, Rui Hou, Song Gao, Danye Zhang, Shulan Zhang, Bei Lin

**Affiliations:** 1 Department of Obstetrics and Gynecology, Shengjing Hospital Affiliated to China Medical University, Shenyang 110004, China

**Keywords:** Ovarian Cancer, Lewis y Antigen, Integrin αv, β3, Chemotherapeutic Drug Resistance

## Abstract

**Objective:**

This study investigates the expression of Lewis y antigen, integrin αv, β3 in epithelial ovarian cancer tissues. We further evaluate the relationship between their expression and chemotherapy resistance of ovarian cancer and its possible clinical significance.

**Methods:**

Tissues of 92 patients with ovarian cancer meeting the inclusion criteria with complete follow-up data were enrolled and divided into chemotherapy resistant group and sensitive group. The expression and relationship of Lewis y antigen and integrin αv, β3 are assessed in paraffin sections using immunohistochemistry and double-labeling immunofluorescence method. Multivariate logistic regression analysis was used to investigate the relationship between age, clinical stage, differentiation, histologic subtype, Lewis y antigen and integrin αv, β3 expression in ovarian cancer patients.

**Results:**

The expression rates of Lewis y antigen and integrin αv in the resistant group, significantly higher than the rates found in the sensitive group (*p* <0.05). Multivariate analysis showed that the expression of Lewis y antigen, integrin αv and ovarian cancer’s clinical stage were independent, drug resistance-related risk factors. The expression levels of Lewis y antigen and integrin αv, β3 were positively correlated with each other.

**Conclusions:**

A close correlation between Lewis y antigen, integrin αv, β3 and ovarian cancer was observed. Lewis y antigen can influence the biological behavior of a tumor cell as an important composition of integrin αv, β3 by some signal pathway. And the expression of Lewis y antigen, integrin αv and ovarian cancer’s clinical stage are both independent, drug resistance-related risk factors.

## Background

Ovarian cancer has the highest mortality rate of all cancers of the female reproductive system. Chemotherapy resistance is an important factor influencing treatment efficacy. In recent years, studies have shown that through the interaction between surface adhesion molecules and surrounding extracellular matrix, tumor cells can promote proliferation, invasion, and metastasis, thus improving their tolerance to chemotherapeutic drugs [1]. Cell adhesion-mediated drug resistance (CAM-DR) is a relatively new theory for the mechanism of drug resistance in tumor cells [2-4]. Integrins comprise a large family of cell adhesion molecules; they are transmembrane glycoproteins that play a key role in the interaction between cells and components of the interstitial space. Integrin-mediated interactions between cells or between cells and the extracellular matrix play an important role in tumor growth, invasion, metastasis, drug resistance, and many other processes [5]. Many studies have confirmed that carbohydrate antigens on the cell surface are closely related to integrins. In our previous work, we have found that as a part of the integrin αvβ3 structure, Lewis y antigen expression is related to the degree of invasiveness of ovarian cancer [6]. Here we use immunohistochemistry to further study the expression of Lewis y antigen and integrin αvβ3 in tissue specimens from patients with chemotherapy resistant or sensitive ovarian cancer and analyze how the expression of these molecules correlates with chemotherapy resistance and the resulting clinical significance.

## Materials and methods

92 chosen paraffin samples are obtained from the operations done from 2006 to 2010 in the department of Gynecology and Obstetrics of Sheng Jing Hospital Affiliated to China Medical University. After the cytoreductive surgery and 6-8 periods of systematic chemotherapy, each patient will receive a follow up observation for at least one year. Among the 92 cases of primary epithelial ovarian cancer studied, there are 58 cases of serous cystadenocarcinoma, 8 mucinous cystadenocarcinoma, 4 endometrioid carcinoma, 7 clear cell carcinoma and 15 poorly differentiated adenocarcinoma. According to histological grade, there were 15 cases of high differentiated, 35 moderate and 42 poor. The group includes 19 cases of stages I, 13 stages II, and 60 stages III (according to International Federation of Gynecology and Obstetrics (FIGO) criteria). All the cases are primary, the information and follow-up data are complete; chemical treatment is not used in all the patients before operations.

### Drug resistance related clinical and pathological parameters

Tissues obtained between 2006 and 2010 from 92 patients with ovarian cancer meeting the inclusion criteria with complete follow-up data were enrolled. The clinical and pathological parameters of ovarian cancer patients include age, clinical stage, differentiation, histologic subtype and chemotherapy scheme (PTX (paclitaxel) + Carboplatin (TC)). According to the guideline of National Comprehensive Cancer Network (NCCN) (recurrence during the chemotherapy period or within 6 months after the chemotherapy was define as drug resistance group; after the chemotherapy recurrence between 6 to 12 months was partial sensitive group and recurrence beyond 12 months after the chemotherapy or didn’t recurrenc was sensitive group), the patients were divided into chemotherapy resistant group (34 cases) and sensitive group (58 cases).

### Main reagents

Mouse monoclonal anti-Lewis y antibody (clone A 70-C/C8) was purchased from Abcam Company (UK). Rabbit polyclonal anti-αv and anti-β3 antibodies were purchased from Santa Cruz (USA). Goat monoclonal anti-rabbit immunoglobulin G fluorescein isothiocyanate (FITC) and goat monoclonal anti-mouse immunoglobulin G tetramethyl rhodamine isothiocyanate (TRITC) were purchased from Fujian Maixin Company (China). DAPI was purchased from Shenyang Baoxin Company (China). Serum albumin (BSA) and DAB kit were purchased from Zhongshan Biotechnology Company (China). Other reagents were supplied by our laboratory.

## Methods

### Immunohistochemistry

Streptavidin-biotin-peroxidase (SP) immunohistochemistry was performed. Tissues were fixed in 4% formaldehyde and embedded in paraffin, and 4 mm thick serial sections were prepared at the same organizational part. The working dilution of Lewis y antibody and integrin αv, β3 antibody were 1:100 and 1:160, respectively. The staining procedure was performed according to SP kit manual. The group with PBS instead of primary antibody was used as a negative control. A colon cancer sample served as positive control for Lewis y antigen, and a breast cancer sample was a positive control for integrin αv, β3.

### Immunofluorescence

The sample slices of strong expression for immunohistochemistry were selected to performed immunofluorescence double labeling method. Primary antibody combinations were anti-integrin αv with anti-Lewis y, or anti-integrin β3 with anti-Lewis y, with the PBS instead of primary antibody as the negative control. The working dilution of rabbit anti-human integrin αv, β3 and mouse anti-human Lewis y antibody were all 1:160. The working dilution of goat anti-rabbit IgM FITC and goat anti-mouse IgG TRITC were 1:100. The working dilution of nuclear dye DAPI was 1:100. The staining was performed according to the instructions of immunofluorescence kit.

### The determination of results

The presence of brown colored granules on the cell membrane or in the cytoplasm was taken as a positive signal, and was divided by color intensity into not colored, light yellow, brown, tan and was recorded as 0, 1, 2, and 3, respectively. We choose five high-power fields in series from each slice, then score them and take the average percentage of chromatosis cells. A positive cell rate of less than 5% was 0, 5 ~ 25% was 1, 26 ~ 50% was 2, 51 ~ 75% was 3, more than 75% was 4. The final score was determined by multiplying positive cell rate and score values: 0 ~ 2 was considered negative (−), 3 ~ 4 was (+), 5 ~ 8 was (++), 9 ~ 12 was (+++). The results were read by two independent observers to control for variability.

Microscopic red fluorescence indicated Lewis y antigen labeled by TRITC, green fluorescence indicated integrin αv, β3 labeled by FITC, while blue fluorescence indicated DAPI-stained nucleus. Pictures of the three individual fluorescence channels were superimposed using image analysis software, with a yellow fluorescence indicated co-localization of Lewis y antigen and integrin αv, β3.

### Statistical analysis

Statistical analyses were performed using the SPSS software Version 11.5. Data expressed as mean ± SD was applied for statistical analysis. The Student’s *t* test was applied to compare data between the two groups, and analysis of variance was applied to compare data among multiple groups.

The Chi-square (χ2) test was applied to analyze the expression of Lewis y antigen, integrin αv, β3 and clinicopathological parameters. The Spearman correlation analysis method was applied to calculate the coefficient R of indexes and to analyze its correlation, A *P* value <0.05 was considered statistically significant.

## Results

### Expression of Lewis y antigen, integrin αv and β3 in different groups

**Figure 1:**
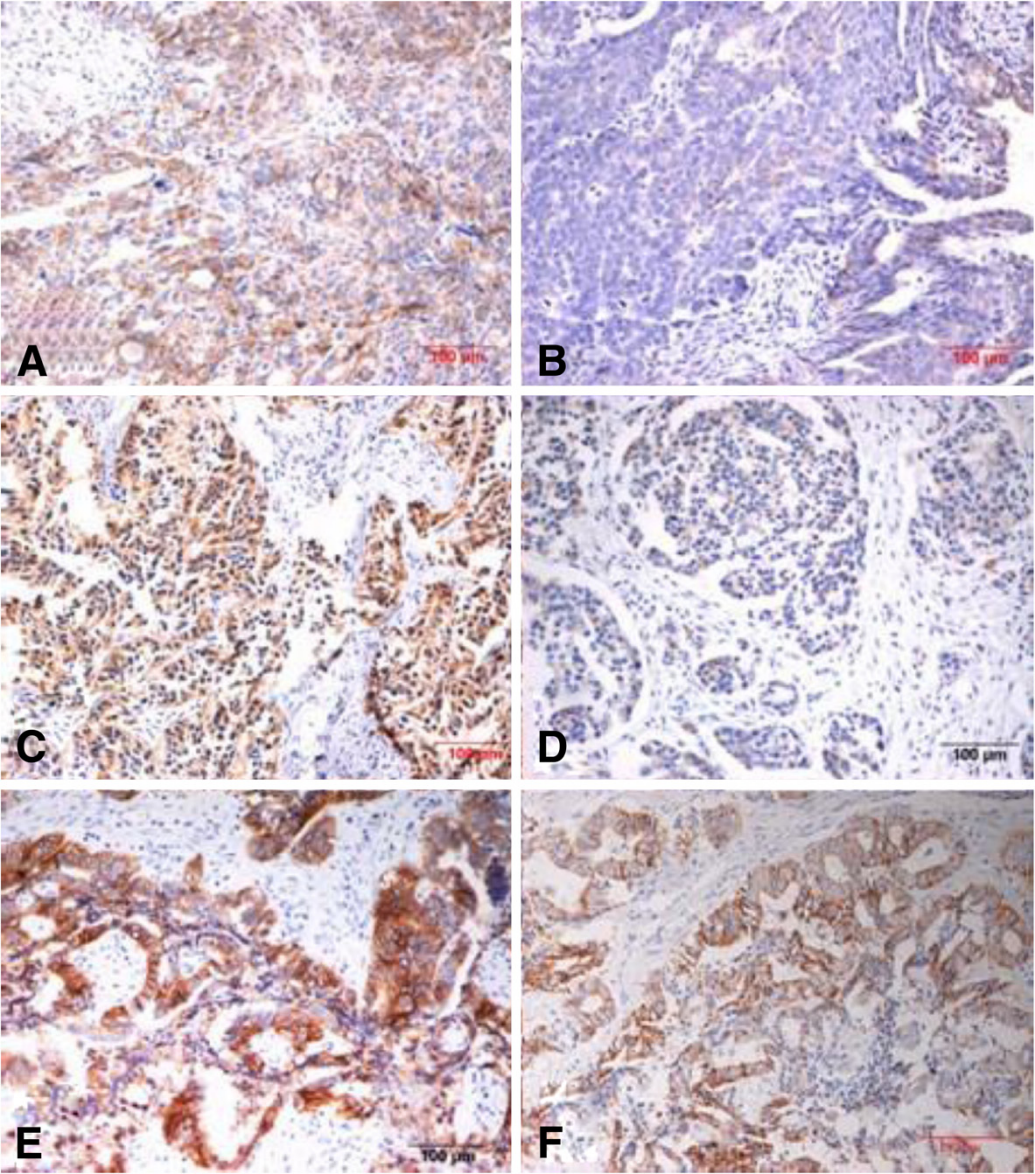
Expression of Lewis y antigen in resistant group (Fig.A: stage IIIc, moderate differentiated serous cystadenocarcinoma) and sensitive group (Fig.B: stage IIIc, poorly differentiated serous cystadenocarcinoma)(*200); Expression of integrin av in resistant group (Fig.C: stage IIIc, moderate differentiated serous cystadenocarcinoma) and sensitive group (Fig.D: stage IIIc, moderate differentiated serous cystadenocarcinoma)(*200); Expression of Lewis y antigen in resistant group (Fig.E: stage IIIc, moderate differentiated endometrioid carcinoma)and sensitive group (Fig.F: stage IIIc, moderate differentiated endometrioid carcinoma)(*200).

Lewis y antigen was expressed in the cytoplasm and cell membrane, mainly on membrane and rarely in the nucleus. The expression rates of Lewis y antigen in the resistant group were 91.67%, significantly higher than 60.34% in the sensitive group (*p* <0.05), as shown in Figure 1 and Table 1.

**Table 1:** Expression of Lewis y antigen in different groups

**Groups**	**Cases**	**Lewis y antigen**				**Positive cases**	**Positive rate (%)**
		**-**	**+**	**++**	**+++**		
Resistant group	34	3	4	19	8	31	91.18
Sensitive group	58	23	16	19	0	36	60.34

Similar to Lewis y, the expression of integrin αv and β3 were mainly on membrane. The integrin αv positive expression rate was 85.29% in the resistant group, significantly higher than that of the sensitive group (51.72%) (*P* < 0.05). The expression rate of integrin β3 in the resistant group was 88.24%, higher than 65.52% in the sensitive group, but there were no significant difference between these two groups (*p* > 0.05), Figure 1 and Table 2.

**Table 2:** Expression of integrin αv and β3 in different groups

**Groups**	**Cases**	**Integrin αv**						**Integrinβ3**					
		**-**	**+**	**++**	**+++**	**Positive cases**	**Positive rate(%)**	**-**	**+**	**++**	**+++**	**Positive cases**	**Positive rate(%)**
Resistant group	34	5	8	11	10	29	85.29	4	10	10	10	30	88.24
Sensitive group	58	28	16	6	8	30	51.72	20	21	15	2	38	65.52

### Drug resistance-related risk factors univariate analysis

The clinical and pathological parameters of ovarian cancer patients include age, clinical stage, differentiation, histologic subtype, only ovarian cancer’s clinical stage were independent, drug resistance-related risk factors (*P* = 0.01), the difference between the rest factors was not significant (*p* > 0.05), as shown in Table 3.

**Table 3:** Drug resistance-related risk factors univariate analysis

**分组**	**Total cases**	**Resistant group**		**Sensitive group**		***P***
		**Cases**	**Rate(%)**	**Cases**	**Rate(%)**	
FIGO Stages						
I	19	1	2.94	18	31.03	0.01
II	13	3	8.82	10	17.24	
III	60	30	88.24	30	51.72	
IV	0	0	0	0	0	
Differentiation						
High	15	5	14.71	10	17.24	0.298
Moderate	35	12	35.29	23	39.66	
Poorly	42	17	50.00	25	43.10	
Histologic subtype						
Serous cystadenocarcinoma	58	24	70.59	34	58.62	0.872
Mucinous cystadenocarcinoma	8	4	11.76	4	6.90	
Endometrioid carcinoma	4	1	2.94	3	5.17	
Clear cell carcinoma	7	1	2.94	6	10.34	
Poorly differentiated adenocarcinoma	15	4	11.76	11	18.87	

### Drug resistance-related risk factors multivariate logistic regression analysis

Multivariate logistic regression analysis was used to investigate the relationship between age, clinical stage, differentiation, histologic subtype, and Lewis y antigen and integrin αvβ3 expression in ovarian cancer patients with ovarian cancer chemotherapy resistance. The result showed that both the expression of Lewis y antigen and integrin αv and ovarian cancer’s clinical stage were independent, drug resistance-related risk factors, as shown in Table 4.

**Table 4:** Drug resistance-related risk factors multivariate logistic regression analysis

**Factors**	**B**	**Sx**	**P**	**OR**	**95% CI**
Lewis y antigen	−2.249	0.605	0.000	0.106	0.032 0.345
Integrin αv	−0.968	0.415	0.020	0.380	0.168 0.857
Clinical stage	−1.304	0.575	0.023	0.271	0.088 0.838

*B*: model parameter.

*Sx*: standard error of mean.

*P*: P value.

*OR*: odds ratio.

**Figure 2:**
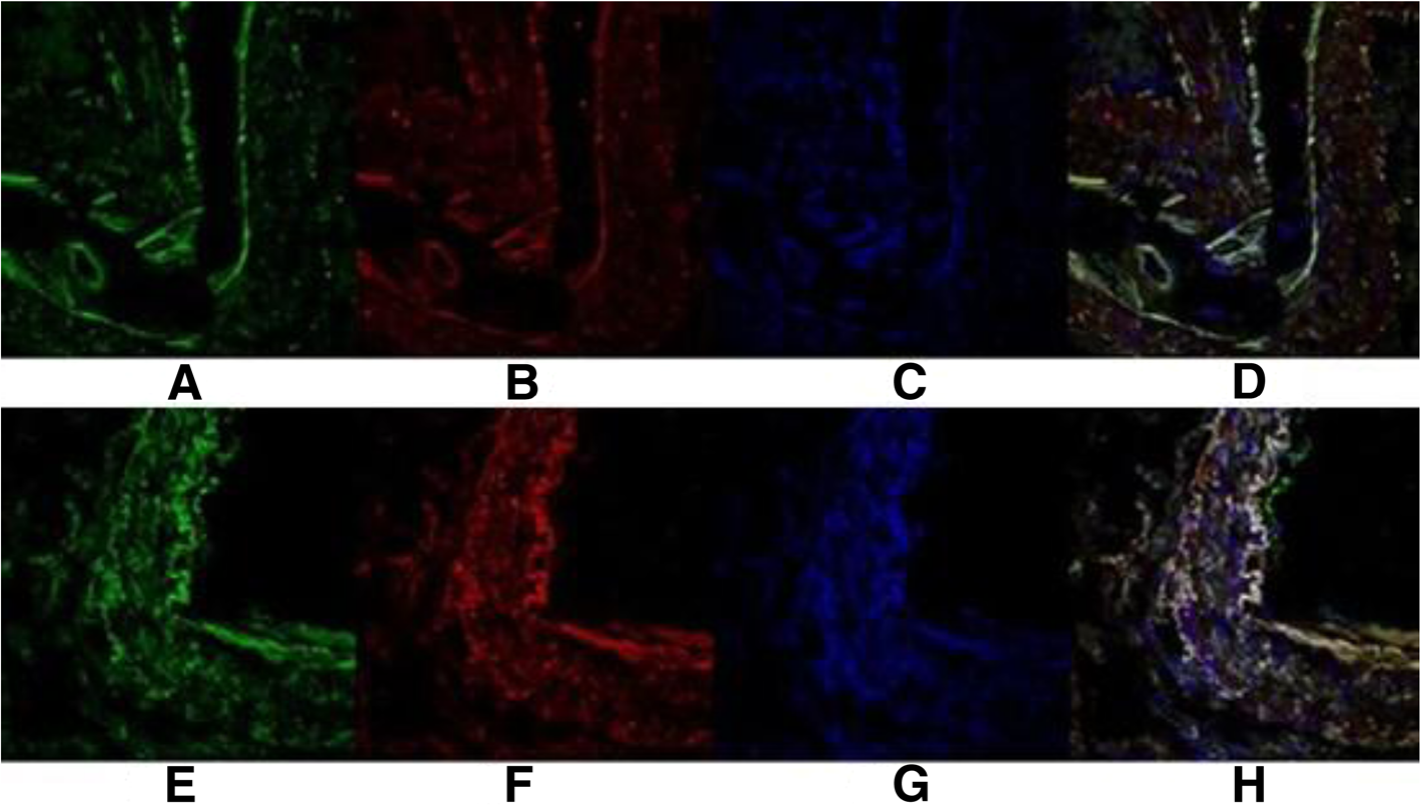
**Integrin αv, β3 and Lewis y colocalize in ovarian malignant tumor.** Integrin αv and β3 (**A** and **E**); Lewis y (**B** and **F**); Nucleus (**C** and **G**); Merged image (**D** and **H**)( *400).

In addition, immunofluorescence double-labeling revealed that in ovarian cancer Lewis y antigen (red fluorescence) was localized in the cell membrane and cytoplasm. Integrin αv and β3 (green fluorescence) were mainly localized in the cell membrane, with a small amount of coloring in the cytoplasm. The 4,6-diamino-2-phenyl indole (DAPI) (blue fluorescence) was used to visualize the nucleus. In three-channel synthesized images, the yellow fluorescence emerges from the area emitting both red and green fluorescence, indicating co-localization of Lewis y antigen and integrin αv, β3, as shown in Figure 2.

### Correlation analysis between expression of Lewis y antigen and integrin αv, β3 in ovarian cancer

A similar trend was seen in the expression of Lewis y antigen, integrin αv, β3 in 92 patients with ovarian cancer, according to the results of immunohistochemistry. Both Lewis y antigen and integrin αvβ3 showed high expression in the ovarian cancer resistant group and their expression levels were positively correlated with each other (Tables 5, 6 Figure 1).

**Table 5:** Correlation between expression of Lewis y antigen and integrin αv in ovarian cancer

**Lewis y antigen**	**Integrin αv**		**Total**
	**Positive cases**	**Negative cases**	
Positive cases	46	20	66
Negative cases	13	13	26
Total	59	33	92

**Table 6:** Correlation between expression of Lewis y antigen and integrin β3 in ovarian cancer

**Lewis y antigen**	**Integrin β3**		**Total**
	**Positive cases**	**Negative cases**	
Positive cases	52	14	66
Negative cases	16	10	26
Total	68	24	92

In order to investigate the correlation between these two antigens, we performed Spearman correlation analysis for each set of data, which demonstrated a high degree of positive correlation between Lewis y and integrin αv (correlation coefficients were 0.235 , *P* < 0.05), β3 (correlation coefficients were 0. 333 , *P* < 0.01 ) subunits.

## Discussion

Chemotherapy resistance has been proven to be a very difficult issue in the treatment of ovarian cancer. The mechanisms of resistance and appropriate countermeasures targeting these mechanisms have become hotspots in ovarian cancer research. Previous studies of the mechanism of resistance in ovarian cancer mainly focused on drug concentration in tumor cells, DNA damage repair mechanisms, glutathione-dependent detoxification enzyme system activity, and other aspects. In recent years, a number of studies on malignant tumor drug resistance have found that tumor drug resistance is related to changes in adhesion molecule composition, the adhesion abilities of tumor cells, and the resultant cytoskeletal rearrangements and signal transduction pathway activation. Therefore, a new mechanism of tumor drug resistance—cell adhesion mediated drug resistance (CAM-DR) has been proposed [2-4].

The adhesion of tumor cells to the surrounding environment can improve cell survival and anti-apoptotic ability. Integrins are important cell surface adhesion molecules as they are receptors for many extracellular matrix components. Integrin receptors can regulate cell growth, differentiation, and metastasis through transmembrane signal transduction. Tumor cell growth and metastasis are both closely related to drug resistance. Metastasized tumor cells are more likely to be drug resistant and resistant tumor cells have a stronger ability to metastasize or invade. The relationship between integrins and drug resistance is gradually gaining recognition, but the research is still in early stages [7-9]. Damiano et al [10] found that the expression of integrin α4β1 in the drug-resistant strain, RPMI8226/S, of human multiple myeloma cell strain RPMI8226 was significantly higher than that in sensitive strains; furthermore, extracellular matrix-coated cells significantly increased the cells’ tolerance of the chemotherapeutic drugs melphalan and doxorubicin and reduced the rate of apoptosis. Similar findings have been observed for leukemia, glioma, breast cancer and small cell lung cancer. In preliminary studies, we have also demonstrated that the ovarian cancer cell line, RMG-I-h, with high expression of the integrins α5β1 and αvβ3, can increase drug resistance to 5-FU, carboplatin, and paclitaxel [11,12].

Integrin glycosylation status has been shown to affect the strength of integrin-ligand binding and the formation of the glycosidic bond catalyzed by glycosyltransferase affecting the glycosylation status of integrins. The fucose sugar has been shown to be a component of integrin molecules. Furthermore, core fucosylation is essential for integrin-mediated cell migration and signal transduction and plays a key role in the interaction between cells and extracellular matrix, thus affecting tumor metastasis. E. W. Easton et al [13] purified α5β1 integrin from human placenta and α3β1 integrin from the uterine epithelial cell line, HCV29, and demonstrated that both integrins were more than 50% fucosylated. Zhao et al [14] found that knockout of the α1,6-fucose transferase gene (FT8) could prevent integrin α3β1-mediated cell migration and cell growth signals, suggesting that core fucosylation is required for the functions of integrin α3β1. Lewis y antigen is an oligosaccharide containing two fucose molecules and falls into the A, B, H, and Lewis blood type families. The role of Lewis y antigen as a cancer-associated antigen in tumorigenesis and development gradually arouses more concern. We have previously demonstrated that the Lewis y antigen is a part of the α5β1 and αvβ3 structures and high expression of Lewis y antigen and integrins α5β1 and αvβ3 can enhance the proliferative and adhesive abilities of cells [6,15]. Furthermore, we have shown We have also previously shown that cell lines and clinical ovarian cancer specimens exhibiting increased expression of Lewis y antigens in integrins α5β1 and αvβ3 are more likely to exhibit a malignant phenotype [6,15,16]. Our studies have also shown that Lewis y antigen can increase the ability of α5β1 and αvβ3 to bind their ligands, fibronectin (FN) and vitronectin (VN), thereby increasing the cells’ resistance to platinum drugs by enhancing cellular adhesion [6,15,17].

On the basis of this body of work, we retrospectively analyzed the expression of Lewis y antigen and integrin αvβ3 in the tissue specimens of patients resistant to platinum drugs and investigated their relationship with drug resistance. We found the rates of expression of Lewis y antigen and αv integrins in the resistant group were significantly higher than those in the sensitive group (*P* < 0.05); however, the expression rate of integrin β3 in the two groups was not significantly different. Multivariate analysis showed that the expression of Lewis y-antigen and integrin αv and the clinical stage of ovarian cancer were both independent drug resistance-related risk factors, suggesting that the detection of Lewis y antigen and integrin αvβ3 could play an important role in the prediction of ovarian cancer patients’ drug resistance, prognosis, and outcome. Correlation analysis showed that Lewis y antigen and integrin subunits αv and β3 in ovarian cancer tissues were highly expressed in ovarian cancer cells and their expression levels were positively correlated with each other. Dual-color immunofluorescence labeling indicated that Lewis y antigen and integrin αvβ3 were co-localized in ovarian cancer tissues, further confirming their correlation of expression.

The adhesion between cells and extracellular matrix can increase the activity of protein tyrosine kinase (PTK), resulting in more tumor cells in G1 phase and phosphoinositide 3 kinase activation [18]. Lewis y antigen is not only a part of the integrin α5β1 and αvβ3 structures, but is also a part of the structure of other adhesion molecules such as CD44 [19]. Therefore, increased expression of Lewis y antigen can improve the adhesion of cells to the matrix and promote cell adhesion and metastasis through corresponding signal transduction pathways. These actions can then enhance cell behaviors that promote malignancy which provides a theoretical basis for altering Lewis y antigen expression and/or downstream signaling modification in the treatment of ovarian cancer. Although the mechanism by which adhesion molecule fucosylation affects drug resistance is not yet clear, it is currently believed that integrin-mediated tumor cell resistance is related to the following factors: (1) regulating apoptosis (Bax/BclX); (2) changing the drug targets (of Topo II); (3) inhibiting DNA injury, and enhancing DNA repair; (4) regulating P27 protein, etc. Our studies have shown that Lewis y-antigen is involved in the aforementioned process, and increases tumor cell drug resistance [15,17]. As a part of the integrin α5β1 and αvβ3 structures, Lewis y antigen can promote the adhesion of integrins to extracellular matrix in order to strengthen focal adhesion kinase (FAK) phosphorylation; increased expression of Lewis y antigen would activate FAK signal transduction pathways, increase cell adhesion, and increase drug resistance by regulating Topo-T, Topo-Iiβ, Bcl-2, and Bcl-XL.

These results suggest that the immunohistochemical detection of Lewis y antigen and integrin αvβ3 in ovarian cancer tissues can be used as important indicators for determining appropriate clinical chemotherapy, prognosis, and outcome. In-depth understanding of signaling transduction pathways for integrin-mediated chemotherapy resistance will provide a basis for increasing chemosensitivity and developing new chemotherapies.

## Competing interests

The authors declare that they have no competing interests.

## Authors’ contributions

JG carried out most parts of the experiment; CL, RH, SG and DZ participated in the experiment; BL and SZ participated in the design of the study; DL and JL performed the statistical analysis; ZH participated in its design and coordination and helped to draft the manuscript. All authors read and approved the final manuscript.
